# Editorial: Structure and function of trans-membrane proteins

**DOI:** 10.3389/fchem.2024.1414079

**Published:** 2024-05-08

**Authors:** Irena Roterman, Michal Brylinski, Fabio Polticelli, Alexandre G. de Brevern

**Affiliations:** ^1^ Department of Bioinformatics and Telemedicine, Jagiellonian University-Medical College, Krakow, Poland; ^2^ Department of Biological Sciences, Louisiana State University, Baton Rouge, LA, United States; ^3^ Department of Sciences, University Roma Tre, Rome, Italy; ^4^ Universite Paris Cite, Universite des Antilles, Universite de la Réunion, Biologie Integree du Globule Rouge, UMR_S1134, BIGR, INSERM, Paris, France

**Keywords:** membrane proteins, protein structure prediction, molecular dynamics, coarsegrained, membranes, hydrophobic mismatch, synchrotron radiation circular dichroïsm, experimental techniques in membrane proteins

Proteins perform the majority of essential biological functions. They can be simplified into 3 main structural classes: globular (i.e., cytosolic), trans-membrane (i.e., embedded in lipid membranes) and fibrous (long and of particular composition, mainly in cytosol). As trans-membrane proteins are embedded in lipid membranes, they are associated with crucial functions such as the passage of essential solutes (ions, carbohydrates, nucleotides, ionic concentration control, etc.) and signaling. The simple support of channel function and the very complex functions of receptors, which control the transport of information signals between the external environment and all the processes taking place inside the cell, make membrane and transmembrane proteins particularly critical for the majority of cellular processes (Pasquadibisceglie et al., 2023a). They are therefore implicated in a large number of pathologies, including many cancers (Pasquadibisceglie et al., 2023b). This explains why 2/3 of drugs target them.

Despite their therapeutic importance, transmembrane proteins remain very mysterious because of their peculiar macromolecular environment, which makes it difficult to obtain experimental data (White, 2023). For example, despite its rapid growth, the protein database currently contains just over 10,000 membrane proteins structures, 20 times fewer than for globular proteins (Kozma et al., 2013). One of the peculiarities of these proteins is the problem of the influence of the external force field on the folding process. Indeed, the polar environment of water pushes hydrophobic residues towards the central zone of the protein, forming a hydrophobic core (Roterman et al., 2023). In contrast, the membrane environment pushes hydrophobic residues towards the protein surface (White and von Heijne, 2004). When membrane proteins are designed to act as transmembrane channels or transporters, the central part of these proteins becomes aqueous, which greatly complicates our understanding (Killian and von Heijne, 2000). Hence, any attempt to elucidate the details of their folding, localization and function is highly anticipated (Gadzała et al., 2019). In this area of modeling, transmembrane proteins have presented particular difficulties (de Brevern, 2010). The advent of deep learning approaches, such as AlphaFold (Jumper et al., 2021), has changed the situation and led to specific developments (Pei and Cong, 2023). In this context, specific dedicated simulation approaches make it possible to generate hypotheses that cannot be obtained experimentally, e.g., (Dabrowska and Brylinski, 2006; Rao et al., 2024). This is why we, Irena Roterman (Poland), Michal Brylinski (United States), Fabio Polticelli (Italy) and Alexandre G. de Brevern (France), decided to launch this Research Topic dedicated to this very passionate class of proteins.

This Research Topic collects scientific contributions concerning the study of trans-membrane proteins, with a specific focus on the use of combined experimental and computational approaches to provide new description of various protein systems.

It seems obvious that transmembrane helices often tend to interact with each other. However, measuring the propensity of transmembrane helices to oligomerise in the plasma membrane is a more complex challenge. Hellmann and Schneider (Hellmann and Schneider) focused on genetic assays that can measure the propensity of transmembrane helices to oligomerise in the plasma membrane of *Escherichia coli* bacteria. They used the dimerisation of the transmembrane helix of human Glycophorin A and its classical mutant (G83I) as a model system. They observed that the addition of leucine residues to the C-terminal helix resulted in pronounced changes in dimerisation propensity using the TOXCAT assay. These results show that topological and structural effects caused by hydrophobic mismatch could further modulate the level of interaction. This point is important because it highlights that the hydrophobic mismatch between the hydrophobic core of a transmembrane helix studied and the membrane of *E. coli* can have an impact on the oligomerization propensity of a transmembrane helix. Thus these TOXCAT calculations should be performed extensively in many more systems.

Bardelang et al. analyzed the expression of virulence genes in the human pathogen *Staphylococcus aureus*. In this system, the quorum-sensing signal molecule agr is an autoinducing peptide generated by initial processing of the pro-peptide AgrD by the transmembrane peptidase AgrB. Little is known about the active form, so the authors used homology modeling and molecular dynamics (MD) annealing to characterize the conformations of AgrB and AgrD in model membranes and in solution. Their analyses reveal a six-helical transmembrane domain (6TMD) topology for AgrB. In solution, AgrD behaves as a disordered peptide that binds N-terminally to membranes in the absence and presence of AgrB. In silico, membrane complexes of AgrD and dimeric AgrB display non-equivalent AgrB monomers responsible for initial binding and processing, respectively. Experimental analyses (fractionated luciferase assay) confirm these results. AgrB and AgrD formed stable complexes in detergent micelles as revealed by circular dichroïsm radiation and Landau analysis. An increased thermal stability of AgrB in the presence of AgrD was evidenced. A conformational change of AgrB upon addition of AgrD was also detected by small-angle X-ray scattering of proteo-detergent micelles. For the first time, an atomistic description of AgrB and AgrD was obtained, as well as confirmation of the topology of the AgrB 6TMD and the existence of AgrBD molecular complexes *in vitro* and *in vivo*.

Floch and collaborators (Floch et al.) have focused on the blood group antigens of the RH system (commonly known as “rhesus”) that have enormous importance in transfusion medicine. They are associated with severe haemolytic consequences of antibodies directed against these antigens, making them the second most important blood group after AB0. A crystal structure of the RhD proteins with their RhAG partner is not available and the exact stoichiometry of the trimeric complex remains unknown. To analyze their structural properties, all combinations of trimers formed by the RhD and/or RhAG subunits were generated by comparative modeling. The conformation of the subunits was relatively constant in the molecular dynamics simulations, with the exception of three large disordered loops. Although only a few differences are observed, this work makes it possible to determine the most likely stoichiometry.

Pasquadibisceglie and collaborators (Pasquadibisceglie et al.) were interested in the ZIP family of proteins (Zrt and Irt-like proteins), which includes transporters responsible for the translocation of zinc and transition metals, such as iron and cadmium between the extracellular space and the cytoplasm, as well as between the cytoplasm and the lumen of organelles. This family of proteins is ubiquitous and ZIP proteins are responsible for the homeostasis of metals essential for cellular physiology. The human ZIP family consists of fourteen members (hZIP1 to hZIP14) divided into four subfamilies. There are common structural features [extracellular domain, the highly conserved transmembrane domain with the binuclear metal center (BMC) and the Histidine-rich intracellular loop], but no comprehensive structural analysis has been performed. Using the AlphaFold2 tool, several structural models were obtained for the 14 hZIP members. It should be noted that specific work on multiple sequence alignments was carried out to refine the quality of these models and to obtain alternative, physiologically-relevant, conformations. A complete three-dimensional model of the hZIP4 homodimer complex was also generated. The inward- and outward-facing conformations obtained suggest that hZIP proteins function by an “elevator-type” mechanism.

Rodríguez-Moraga and collaborators (Rodríguez-Moraga et al.) have analyzed the effect of Rhamnolipids (RLs) on fungal membrane models as described by their interactions with phospholipids and sterols. RLs are secondary metabolites naturally produced by bacteria of the genera *Pseudomonas* and *Burkholderia*; they have biosurfactant properties. They may be of interest for crop protection due to their direct antifungal and eliciting activities. Because they have amphiphilic moieties, it has been hypothesized that they interact directly with membrane lipids and that this may even control the subsequent activity of the RLs. To evaluate this hypothesis, molecular dynamics simulations were carried out. The results suggest direct insertion of RLs into bilayers just below the lipid phosphate groups. This arrangement leads to membrane fluidization. In addition, the acyl chains involved in the interactions may be essential for the membranotropic biological actions of RLs, adhering to the ergosterol structure and forming a significantly higher number of van der Waals contacts than is observed for the acyl chains of phospholipids. All of these interactions may be critical for the biological actions of RLs in this context.

In conclusion, contributions included in this Research Topic highlight the complexity of the structures, interactions and physiological actions of transmembrane proteins, and how a combination of advanced computational simulations and experimental studies can help elucidate this complexity.
